# Mental Health Professionals’ Attitudes Toward Digital Mental Health Apps and Implications for Adoption in Portugal: Mixed Methods Study

**DOI:** 10.2196/45949

**Published:** 2023-06-02

**Authors:** Diogo Nogueira-Leite, José Miguel Diniz, Ricardo Cruz-Correia

**Affiliations:** 1 Department of Community Medicine, Information and Decision in Health Faculty of Medicine, University of Porto Porto Portugal; 2 Nova School of Business and Economics Health Economics and Management Knowledge Center New University of Lisbon Lisbon Portugal; 3 Programme in Health Data Science Faculty of Medicine, University of Porto Porto Portugal; 4 Center for Health Technology and Services Research Porto Portugal

**Keywords:** mobile health, mHealth, health policy, mental health, digital health, apps, psychiatrists, psychologists, technology acceptance, adoption, government regulation

## Abstract

**Background:**

Digital health apps are among the most visible facets of the ongoing digital transition in health care, with mental health–focused apps as one of the main therapeutic areas. However, concerns regarding their scientific robustness drove regulators to establish evaluation procedures, with Germany’s Digitale Gesundheitsanwendungen program pioneering in app prescription with costs covered by statutory health insurance. Portugal gathers a set of conditions and requirements that position it as an excellent test bed for digital health apps. Its daunting mental health landscape reinforces the potential interest in new interventions. To understand if they would be acceptable, we need to understand the supply side’s attitudes and perceptions toward them, that is, those of psychiatrists and psychologists.

**Objective:**

This study aims to understand the attitudes and expectations of psychiatrists and psychologists toward digital mental health apps (DMHAs) in the Portuguese context, as well as perceived benefits, barriers, and actions to support their adoption.

**Methods:**

We conducted a 2-stage sequential mixed methods study. Stage 1 consisted of a cross-sectional web survey adapted to the Portuguese context that was delivered to mental health professionals and psychologists. Stage 2 complemented the insights of the web survey results with a key opinion leader analysis.

**Results:**

A total of 160 complete survey responses were recorded, most of which were from psychologists. This is the most extensive study on mental health professionals’ attitudes and perceptions of DMHAs in Portugal. A total of 87.2% (136/156) of the respondents supported the opportunity to prescribe DMHAs. Increased health literacy (139/160, 86.9%), wider adherence to treatment (137/160, 85.6%), and proper disease management (127/160, 79.4%) were the most frequently agreed upon benefits of DMHAs. However, only less than half (68/156, 43.6%) of the respondents planned to prescribe or recommend DMHAs, with psychologists being more favorable than psychiatrists. Professionals faced substantial barriers, such as a lack of information on DMHAs (154/160, 96.3%), the level of initial training effort (115/160, 71.9%), and the need for adjustments of clinical processes and records (113/160, 70.6%). Professionals reported that having more information on the available apps and their suitability for health objectives (151/160, 94.4%), more scientific evidence of the validity of the apps as a health intervention (147/160, 91.9%), and established recommendations of apps by specific clinical guidelines or professional societies (145/160, 90.6%) would be essential to foster adoption.

**Conclusions:**

More information about DMHAs regarding their clinical validity and how they work is necessary so that such an intervention can be adopted in Portugal. Recommendations from professional and scientific societies, as well as from governmental bodies, are strongly encouraged. Although the benefits of and the barriers to using these apps are consensual, more evidence, along with further promotion of mental health professionals’ digital literacy, is needed.

**International Registered Report Identifier (IRRID):**

RR2-10.2196/41040

## Introduction

### Digital Health Apps

Digital health apps substantiate, perhaps more tangibly than most solutions developed so far, the opportunities the digital age may bring for human health [1-3]. Chief among them is the potential to make health care simultaneously more accessible and personalized. The magnitude of the business-to-consumer market speaks for itself; according to IQVIA’s Digital Health Trends 2021 report [4], >350,000 health apps are available in various app stores, with 110 apps downloaded >10 million times and accounting for approximately 50% of all downloads.

The same report [4] highlights that the COVID-19 pandemic gave a solid impulse to app use, with mental health, cardiovascular disease, and diabetes management–related apps accounting for approximately 50% of disease-focused offering in app stores. The number of downloads and the variety of apps allowed many to conclude that there is an apparent demand at the consumer level. Simultaneously, patients’ perspectives on app use in settings where prescriptions are available [5,6] reflect that patient acceptance still has a long way to go, although some evidence on how to enhance it is already available.

### Regulatory Approaches

The realization that most tools in the field needed to be more mature to match formulated expectations prompted specialists to increasingly voice concerns that most apps need to meet more clinical and technical validation standards, often lacking any empirical support for their adoption [7-11]. A growing consensus is that regulation is needed, especially for apps that diagnose, treat, or manage high-risk conditions [12,13]. Governments and regulators have started to define policy frameworks to determine the benefits of digital solutions [14-18], trying to understand ways to reduce uncertainty around digital health app use and kick-start discussions on their potential payment or reimbursement.

Germany took the lead by implementing a regulatory framework specific to digital health apps and their market access and reimbursement. Its Digitale Gesundheitsanwendungen (DiGA) program [19] was inaugurated in October 2020, and on January 23, 2023, a total of 40 apps qualified for statutory insurance reimbursements. Of these 40 apps, 18 (45%) were classified as DiGA for mental disorders [20]. France is studying a replication of the DiGA approach and has a preliminary reimbursement process through its assessments of medicotechnical and medical benefits [21,22].

Belgium ranks second in implementation; although mHealthBelgium [23] was launched in 2018, it officially started conducting appraisal and reimbursement processes in January 2021, with its selection process based on a 3-level validation pyramid [24]. Most European countries have so far opted for softer, more decentralized approaches, with legal obligations and compliance rules based on the General Data Protection Regulation [25] or the Medical Devices Regulation [26]. By contrast, Singapore and the United States resort to their medical device regulations. The Food and Drug Administration has been particularly active, basing its assessment of apps and digital therapeutics on the Software as a Medical Device framework [27,28]. In September 2022, it updated its *Policy for Device Software Functions and Mobile Medical Applications* [29] and its guidance on clinical decision support software [30], divulged its key findings from the precertification pilot program at the federal level [31], and launched its *Digital Health Policy Navigator* for developers [32].

### Promise of Digital Mental Health Apps to Aid Care Delivery

Mental disorders are one of the areas where the penetration of digital health apps is most prevalent [4,20]. Reasons for their apparent popularity range from the stigma of seeking treatment and individual privacy needs to the convenience of doing it from everywhere and the diversity of treatments available (eg, meditation, cognitive behavioral therapy, group therapy, teleconsultation, etc) [33-38]. These disorders are also one of the disease areas in desperate need for increased and enhanced access. This need already existed before the COVID-19 pandemic, and many have pointed to the deleterious impact of the pandemic on mental health as one of its considerable long-term consequences [39-41]. The burden of disease it entails, both before and after the COVID-19 pandemic, and the way it impacts many other health conditions make it a priority for action [42-44].

Portugal is often cited as a country where mental disorders, particularly anxiety and depression, are above average; the prevalence of mental health disorders in 2019 was estimated at 8.27% of disability-adjusted life years and 19.27% of disease cases. The statistics for anxiety and depressive disorders were expected to be 2.58% and 3.16% of the total disability-adjusted life years and 9.08% and 5.88% of the disease prevalence, respectively [45]. A summary of its comparison with the global, European Union (EU), and German landscape is presented in Table 1.

Conversely, there is limited access to psychological and psychiatric care, with waiting times ranging from 13 to 237 days for a psychiatry consultation in the Portuguese National Health Service from July to September 2022 [46]. The time frame for a psychology consultation in the 11 institutions that reported it for the same period ranged from 15 to 134 days. Considering most depression and anxiety cases, albeit responsible for most of the disease prevalence of mental disorders, are classified as nonpriority cases, waiting times can be expected to range from 44 to 237 days.

In a country burdened by out-of-pocket payments about double the EU average [47,48], the possibility of resorting to private sector providers is minimal, as insurers cover only some associated costs. It is necessary and urgent to find new solutions. The combination of disease prevalence and lack of access to care, along with a relatively digitized health system and average indicators of digital literacy, makes Portugal an excellent test bed to understand whether digital mental health apps (DMHAs) can, or cannot, help people receive the care they need.

One of the key promises of digital health apps is increased access. However, no innovative intervention in health—be it a drug, medical device, or any other—achieves critical mass without the endorsement of health professionals [49-51]. Therefore, it becomes essential to understand, from the perspective of mental health professionals (here defined as psychologists and psychiatrists), their level of comfort with digital health apps, their main challenges in adopting them, and what can enable and enhance their use.

To our knowledge, only one study has been performed on the Portuguese landscape of web-based interventions for psychologists [52]. No studies were found concerning the attitudes of psychiatrists in Portugal or combining the attitudes and perspectives of Portuguese psychiatrists and psychologists toward DMHA as a specific web-based intervention. Our study aimed not only at bridging these gaps but also at contributing importantly to do so (1) after the COVID-19 pandemic and its catalyzing effect on telehealth adoption [53]; (2) after major prescription and reimbursement processes were enacted in the EU space; and (3) by mapping the supply side of web-based mental health care, given the mediating effect of mental health professionals [50]. Our study contributes substantially to researchers, academia, industry, and policy makers by providing necessary information on how to leverage the DMHA as a tool to increase access to mental health care and improve patient outcomes while reducing the burden of disease associated with mental health disorders.

**Table 1:** Share of disability-adjusted life years (DALY) and disease prevalence (in percentage points) per condition and geography. Data source: Institute for Health Metrics and Evaluation (IHME). GBD Compare Data Visualization. Seattle, WA: IHME, University of Washington, 2020.

	Portugal			Germany			EU^a^			World	
	DALY (%)	Prevalence (%)	DALY (%)		Prevalence (%)	DALY (%)		Prevalence (%)	DALY (%)		Prevalence (%)
Mental disorders	8.27	19.27	6.43		15.59	6.65		15.34	4.92		13.04
Depression	3.16	5.88	2.16		4.32	2.42		4.6	1.84		3.76
Anxiety	2.58	9.08	1.95		7.07	1.69		5.82	1.13		4.05

^a^EU: European Union.

### Objective

This paper aimed to understand mental health professionals’ attitudes (defined as psychologists and psychiatrists in this study) toward DMHAs in the Portuguese context. Mental health professionals will be questioned regarding perceived benefits, barriers to adoption, and potential ways of supporting the adoption of DMHAs. These apps were the focus of this study. The authors aimed to achieve this by directly inquiring mental health professionals regarding their specific clinical practices, perceived needs, and expectations.

## Methods

### Study Design

The research team used a mixed methods methodology. Stage 1 consisted of a cross-sectional web-based survey adapted to the Portuguese context and delivered to mental health professionals and psychologists. It used a web-based quantitative data–focused survey, adapted to the Portuguese context, which served as a primary data source. Stage 2 used the answers collected from the survey to help conduct a qualitative key opinion leader (KOL) analysis.

As per the research protocol [54], the methods initially intended for this study had to be adapted because of the survey’s low response rate. Both the web-based survey and the structure of the KOL analysis followed the same constructs studied by Dahlhausen et al [55] to maximize comparability with that study and the German landscape, albeit focused on mental health.

Notably, this study did not include a literature review of technology adoption, relevant case studies, or subsequent interviews with mental health professionals and psychologists on their views and perceptions toward DMHAs. This was deemed appropriate, as such processes had the objective of building up the questionnaire, and we intended to apply a translated version of the survey to the Portuguese context. Following the original publication, we conducted a web-based survey on a pretest group of health care professionals.

In Portugal, psychiatrists and psychologists are expected to prescribe or otherwise interact with DMHAs and act upon the patients’ mental health. To maximize the targeting of these professionals, we restricted our approach to these 2 groups of health care workers. Moreover, to complement our interpretation of the survey results and help us understand the meaning and generalizability to the national context, we conducted a KOL analysis with a select set of professionals belonging to 1 of the 2 surveyed groups, with roles in clinical practice, academia, industry, or a combination of these.

Furthermore, given that no prescription processes are established in Portugal for DMHAs, it is not possible to rigorously define who would be authorized to recommend or prescribe DMHAs. Therefore, we asked clinicians to answer questions that report to recommendation or prescription according to their own cases, as psychiatrists are allowed to prescribe medication in Portugal, whereas psychologists are not. Our results should be interpreted accordingly.

Moreover, in our survey, we did not ask about health insurance coverage status, as it proves more relevant, in the Portuguese context, to understand whether they work for the National Health Service, in private practice, or both. As previously stated, we targeted only psychiatrists and psychologists for this survey, with the latter comprising most of the respondents (127/158, 80.4% of the answers). Although no data regarding the number of psychologists are available at the time of this study’s conclusion, it is our perception and that of the KOLs that the largest share of mental health professionals would be attributable to this group of practitioners.

In addition, we chose to represent survey data differently, intending to highlight the distribution of the categorical (Likert scale) answers and define their centrality without recurring to arithmetic operations.

### Web-Based Survey Design

The first part of the study comprised a cross-sectional, web-based survey. We used the final survey questionnaire available in the Multimedia Appendix 1 in the study by Dahlhausen et al [55] as given and translated it to Portuguese using a licensed translator (Multimedia Appendix 2).

This translation was delivered to 10 mental health professionals—5 from each professional group, psychologists and psychiatrists—to gather their input. Mental health professionals’ feedback was focused on calibrating the survey to (1) reflect essential questions to ask regarding the use of digital health tools by mental health professionals and (2) adapt to a Portuguese mental health care context. This allowed us to focus solely on mental health and DMHAs. The survey used by Dahlhausen et al [55] depicted, although implicitly and more pragmatically, the theoretical constructs of the Unified Theory of Acceptance and Use of Technology [56]. Given that our adaptation process did not affect this, we considered our questionnaire, by the same token, to adapt to the same theory and its constructs. The obtained feedback was incorporated to produce a final survey questionnaire for this study, available in English in Multimedia Appendix 1. Therefore, several changes were made, including modifying and adding questions, per the survey reviewers’ suggestions. Although these limit the direct comparability between studies, they reflect the different needs and issues of the 2 countries. Both translations—the questionnaire by Dahlhausen et al [55] to Portuguese for adaptation and the final adapted survey questionnaire in Portuguese to English—were performed by SPS Traduções, a specialized translation firm.

Before broad diffusion, the survey questionnaire was pretested by 5 different colleagues to determine the completion time and identify shortcomings. As a result, an introductory page on digital health apps and developments in their regulatory landscape was included to provide initial baseline information before the start of the survey. The completion time was estimated to be between 4 and 7 minutes. To establish a basis for comparison with a reimbursable app system, mental health professionals were asked to consider a scenario in which these apps fulfilled regulatory requirements and addressed safety, quality, and efficacy concerns. Accordingly, mental health professionals’ responses are to be interpreted under this assumption and not necessarily to these apps’ current form as available in Portugal. Nonetheless, it could be argued that because both Portugal and Germany belong to the EU and its internal market, an app developer would want to maximize comparability between apps, tweaking them for populational specificities.

Several web-based channels and methods were used to distribute the questionnaire to health care professionals. The survey’s link was circulated in the newsletter of the Portuguese Order of Psychologists and through the social media of several members of the Psychiatry Specialty College of the Portuguese Order of Medical Doctors. The professionals who engaged in the questionnaire’s adaptation were invited to perform snowball recruiting by sharing the survey link through their social media accounts and with professional contacts and forums where they were involved.

In addition, Knok healthcare [57], a fully integrated telemedicine platform company, offered to disseminate the questionnaire on its social media accounts to its relevant audience of health care professionals. This free initiative is part of Knok’s mission to deliver social impact by divulging the potential benefits of telemedicine. Finally, the Portuguese Society of Psychiatry and Mental Health agreed to disseminate the survey via social media on Twitter.

The platform used was Inqueritos@UP, the University of Porto’s internal survey manager by LimeSurvey. The survey adhered to and was reported following the Checklist for Reporting Results of Internet E-Survey (CHERRIES) guidelines. The period for answer collection ran from September 26, 2022, to November 6, 2022, the same 6-week period applied in the study by Dahlhausen et al [55].

**Figure 1:**
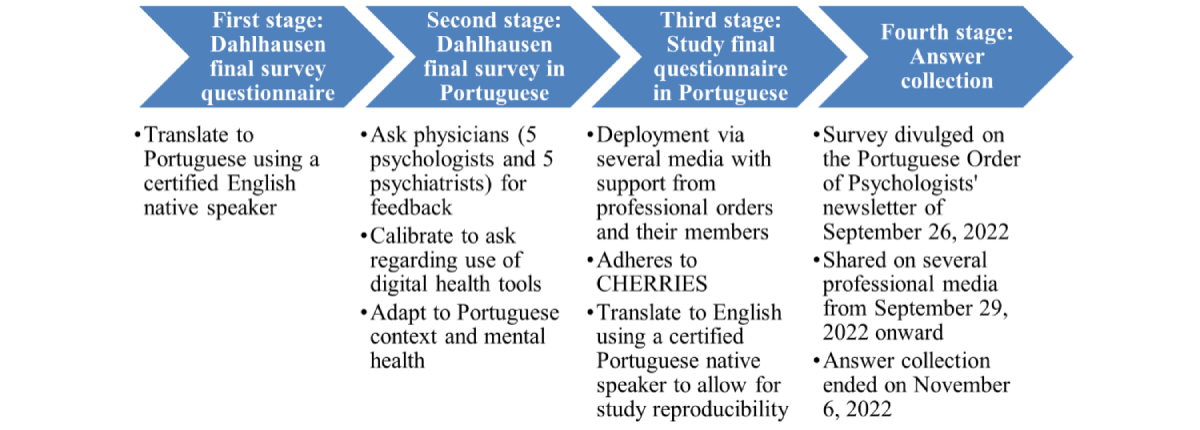
Survey adaptation and communication workflow. CHERRIES: Checklist for Reporting Results of Internet E-Survey.

The study’s Data Protection and Privacy Policy, made available in the *Ethics Approval, Informed Consent, and Participation* section, comprises all relevant information on these aspects. To maximize responses, the only inclusion criterion was to be registered with the mental health professional’s respective professional order. No exclusion criteria were introduced, and no financial incentives were offered. Figure 1 summarizes the survey’s adaptation and communication workflow.

The gathered data were analyzed according to the methods used in the study by Dahlhausen et al [55] to allow for maximum comparability between the results. Descriptive statistical analyses were performed for all variables, whenever possible. Estimates of association for the variables corresponding to “Results” subsections in the study by Dahlhausen et al [55] were also computed.

Only data excluded because of different health system organizations and their consequences for mental health professionals (eg, statutory health insurance in Germany vs little to no point-of-care payments in Portugal) or of reasonable suggestion during the feedback period were treated differently and according to the nature of each variable.

The correspondence map between the initial questionnaire (ie, by Dahlhausen et al [55]) and the final survey questionnaire is presented in the table in Multimedia Appendix 3 [54].

Furthermore, data were analyzed to find associations among variables, especially between health care professionals’ demographic and professional characteristics, attitudes toward DMHAs, and the likelihood of prescription. These were conducted on RStudio (version 2022.07.1 build 554; RStudio Inc) using chi-square tests or, when conditions for using chi-square tests were not met, Fisher exact tests with Monte Carlo approximation and 2000 replicates [58,59]. R packages used for data processing, analysis, and graphical representations were *tidyverse*, *data.table*, *png*, *gt*, *gtExtras*, *gtsummary*, *Hmisc*, *likert*, *grid*, *forcats*, *scales*, *reshape2*, and *rcompanion*.

### KOL Analysis

The KOL analysis [60] served two purposes: (1) to compile what the prominent opinion voices in psychiatry and psychology in Portugal and across academia, clinical practice, and industry understand to be the main benefits, adoption barriers, and measures that can support the adoption of DMHAs and (2) to gather their input on the conducted survey’s results to understand whether they agree with their perception of most Portuguese psychiatrists’ and psychologists’ views on DMHAs.

The KOL analysis followed a 2-step approach. The first step consisted of semistructured individual web-based interviews, followed by a second round of confirmation of the gathered consensus. The method used for the KOL analysis could be defined as a compromise between the Nominal Group Technique and the Delphi Technique [61].

In the first part of this interview, each KOL was asked what were, in their opinion, the top 3 benefits, barriers to adoption, and measures that could support the adoption of DMHAs in the Portuguese context. In the second part of the interview, the interviewers showed KOLs the results of the conducted web survey and asked them to comment on the results. The requested comments were focused on whether these results agreed with their perception of most Portuguese psychiatrists and psychologists, asking them to justify their opinions and statements.

The second stage of the KOL analysis consisted of circulating the main elements gathered during performed interviews and asking for their comments in free text, namely, whether they agreed with the established consensus and whether something important was missing. This analysis was divided into benefits, barriers to adoption, and support measures to ensure methodological consistency.

A total of 25 KOLs were identified and selected to participate in this research (practicing psychiatrists or psychologists, researchers, and managers with a psychiatry or psychology background working in digital health companies). Invitations to participate were made via email through the identification of publicly available professional email addresses. Snowball recruiting was used to find more participants; every contacted KOL was asked to suggest other KOLs that could be reached for this study. KOLs were given a 7-day period to answer whether they wanted to participate and, if so, to point to 3 dates and times for the interview. Those who did not respond to the initial invitation received a follow-up email after 3 days to increase the response rate. Interviews ran from November 25 to December 23, 2022, lasting between 35 and 70 minutes.

Overall, 4 of the KOLs were psychiatrists and 2 were psychologists. This is an important feature to remember, as the number of psychiatrists who answered the survey was much smaller than that of the psychologists. This allowed us to expand the interpretation power of the answers provided by psychiatrists. Notably, every KOL contributes regularly to the public discourse on mental health and the use of technology to tackle problems around mental ill health, for example, in written media. Moreover, half of the KOLs (3/6, 50%) have already developed DMHAs or more comprehensive digital health tools, and 5 (83%) out of 6 KOLs work in academia and private or public sector.

### Ethics Approval, Informed Consent, and Participation

The Ethics Committee of the Faculty of Medicine of the University of Porto pronounced itself favorable to the research project on June 30, 2022 (Opinion 52/CEFMUP/2022).

Ethical considerations and safeguards for the study and its supporting documents (including the web-based survey) were encoded in the study’s Data Protection and Privacy Policy, which received approval from the Data Protection Officer of the University of Porto and is transcribed as follows:

- To preserve participants’ privacy, they will not be asked to provide any personally identifiable information. In addition, participants will not be tracked for having started or completed the survey, increasing privacy but limiting the possibility of reminders.
- *Informed consent and consenting capacity*: all potential participants (mental health professionals and academic community members) will be given web-based written information on the study and its objectives and will be asked to provide consent (click to agree) that they are willing to participate, do so freely and voluntarily. Nonparticipation will not compromise their current roles. Participation in the study will be voluntary, and no inducements or incentives to participate will be offered.
- *Confidentiality*: Any data or personal details that could potentially reveal the identity of individuals will be removed. Only anonymized, deidentified information will leave the place of origin. A database with responses will be maintained on a password-protected database. All research data will be stored on a password-protected desktop computer at the host organization. Study participants will be invited, through a link provided on the last page of the survey, to provide their name and electronic address to allow the research team to facilitate their receipt of a synopsis of the study findings on publication. This list will be kept separately on a password-protected database and a password-protected desktop computer at the host organization. All data will be stored securely at the host institution and destroyed 3 years after the PhD defense date. It is estimated that the PhD will be defended between October 2023 and December 2023.
- General Data Protection Regulation compliance will be adhered to in terms of the following:
  Data privacy rights: participants will have the right to request information about their data throughout the research process.
  Transfer of data: participants will be informed about the circumstances under which their data may be transferred and safety measures that will be taken to protect the data (eg, data are encoded).
  Retention of data: Participants will be informed of the duration for which their data will be stored.

Using Inquéritos@UP, survey data were stored at the university’s servers and thus not shared with external entities, constituting another layer of privacy protection. Furthermore, the survey’s first page briefly explained the required data and the rationale behind it.

## Results

### Web-Based Survey

#### Demographics

A total of 160 health care professionals completed the questionnaire, with only some nonresponses to specific questions. Although the overall survey response rate could not be determined, given its means of distribution and the adopted privacy-ensuring settings, 400 people opened the survey. This translates into a completion rate of 40%, making this study the most extensive on mental health care professionals’ attitudes and expectations toward DMHAs in Portugal.

Table 2 shows the characteristics of those who completed the questionnaire and their work. The most common age group was the 36-45 years segment (59/160, 36.9%), closely followed by the 26-35 years segment (56/160, 35%), being skewed toward a younger population. Of the 160 participants, 134 (83.7%) participants were female, likely presenting a higher representation in the sample than in the national presentation (52.8% of all psychiatrists in Portugal were female, with no publicly available data for psychologists) [62]. Most respondents (136/160, 85%) served populations with >20,000 residents, representing a primarily urban setting.

Many practitioners worked in >1 type of practice, most commonly at clinics (57/160, 35.6%) and hospitals (56/160, 35%), with only 10.6% (17/160) working at the primary care level. A considerable portion of professionals were involved with private consultation services, either individually (35/160, 21.9%) or in a group (39/160, 24.4%). However, in the study by Dahlhausen et al [55], most clinicians were split between single or joint practice environments. Among those who answered about the number of mental health professionals and psychologists they worked with, most (64/119, 53.8%) reported having ≥5 such professionals in their workplace, with 26.9% (32/119) of participants reporting >10 professionals in their workplace. Of these 160 participants, 127 (79.4%) were psychologists and 25 (15.6%) were medical psychiatrists. The distribution of answers was relatively homogeneous with regard to the number of mental health professionals in the workplace. In the replicated paper, approximately half of the respondents (613/1268, 48.3%) reported having only 1 practitioner.

**Table 2:** Demographic and professional characteristics of the sample (N=160).

Characteristics			Values, n (%)
**Age (years)**			
	<26	4 (2.5)	
	26-35	56 (35)	
	36-45	59 (36.9)	
	46-55	31 (19.4)	
	56-65	6 (3.8)	
	>65	4 (2.5)	
**Sex**			
	Female	134 (83.8)	
	Male	26 (16.2)	
**Size of population covered (inhabitants)**			
	<5000	5 (3.5)	
	5001-20,000	0 (0)	
	20,001-100,000	36 (25.5)	
	100,001-500,000	52 (36.9)	
	>500,000	48 (34)	
	Unknown	19 (11.9)	
**Workplace**			
	Hospital	56 (35)	
	Primary care	17 (10.6)	
	Clinic	57 (35.6)	
	Individual private consultation	35 (21.9)	
	Collective private consultation	39 (24.4)	
**Number of doctors and psychologists in workplace**			
	1	19 (16)	
	2	15 (12.6)	
	3	13 (10.9)	
	4	8 (6.7)	
	5	10 (8.4)	
	6	6 (5)	
	7	5 (4.2)	
	8	4 (3.4)	
	9	4 (3.4)	
	10	3 (2.5)	
	>10	32 (26.9)	
	Unknown	41 (25.6)	
**Profession**			
	Psychologist	127 (79.4)	
	Psychiatrist	25 (15.6)	
	Other	6 (3.8)	
	Unknown	2 (1.3)	

#### Perceived Potential Benefits From DMHAs and Attitudes Toward DMHAs

**Figure 2:**
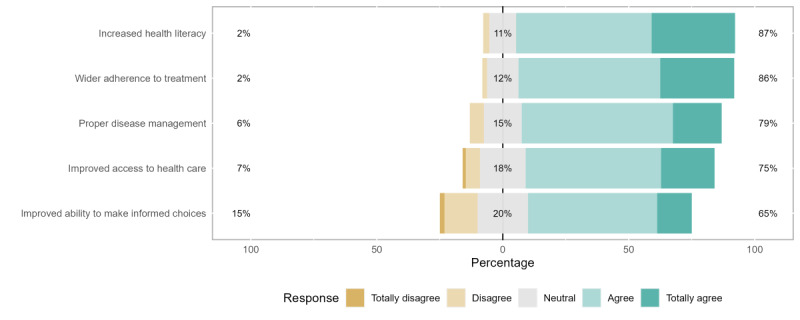
Perceptions of potential benefits for patients.

**Figure 3:**
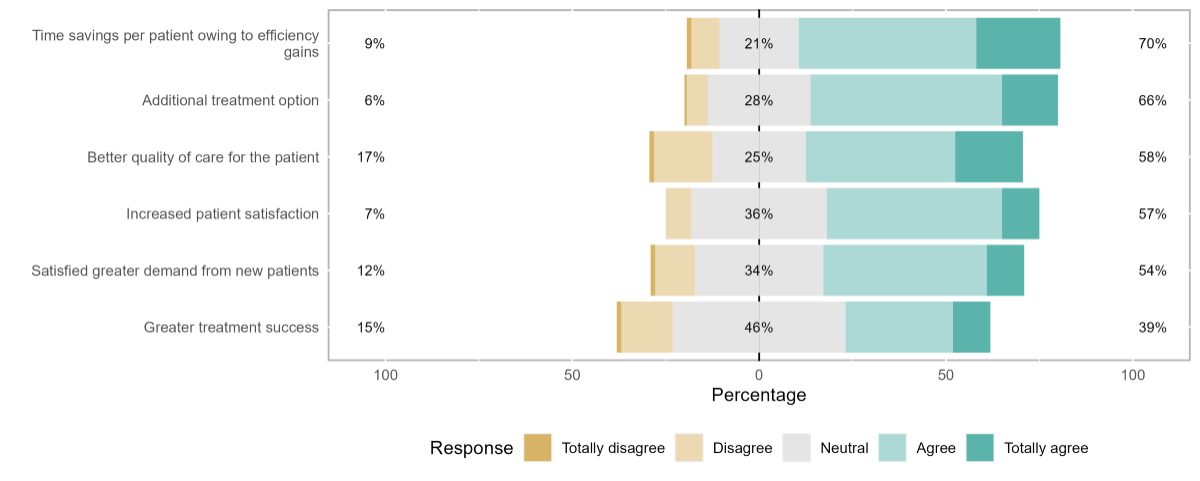
Perceptions of potential benefits for health care professionals.

**Figure 4:**
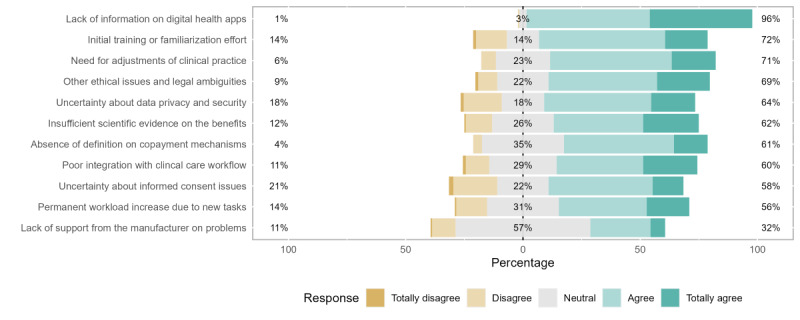
Perceived barriers to prescription.

**Figure 5:**
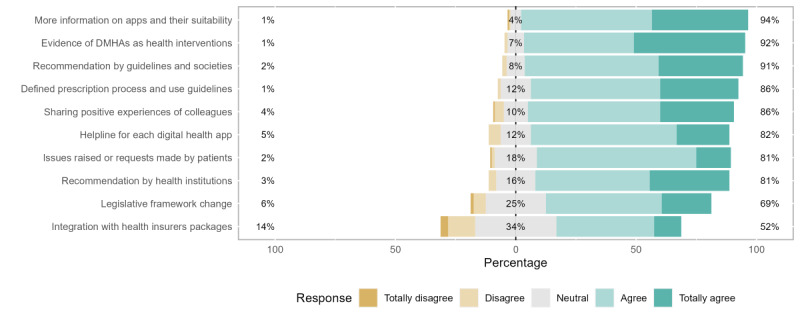
Measures to support adoption. DMHA: digital mental health app.

Figure 2, as it happens to the following figures (ie, Figures 3-5), provides a visual representation of the Likert scale responses, ordered according to the color-coded legend. For each question (eg, increased health literacy), an overall share is represented for negative (“Totally Disagree” or “Disagree”), neutral (“Don’t Know” or “Neither Agree nor Disagree”), or positive (“Agree” or “Totally Agree”) answers. Their percentage is displayed on the left, central, and right positions of the stacked bars, respectively. As far as central tendency measures are concerned, we selected the median value of each group of answers besides the corresponding bar. The neutral response group was set as the center of the axis to facilitate comparisons between answers, with distribution graphs representing the distribution of the provided answers.

Potential benefits to the patient from DMHAs, namely, improved ability to make informed choices, proper disease management, improved treatment adherence, improved access to health care, and increased health literacy were perceived very positively by responding health professionals. This was demonstrated by the overall positive perceptions toward using DMHA services; in no case did the general agreement have <65% of the responses. A higher general agreement proportion of the answers (including “Agree” and “Totally Agree”) was found concerning the gains in health literacy (139/160, 86.8%) and treatment adherence (137/160, 85.6%).

With regard to general disagreement ratios (including “Disagree” and “Totally Disagree”), the most unfavorable perceptions (24/160, 15%) were demonstrated toward the capacitation of informed choices by the patients. This question (32/160, 20%) and another regarding improved access to health care (29/160, 18.1%) represented the highest proportions of neutral answers (including “Don’t Know” and “Neither Agree nor Disagree”).

In parallel, the same evaluation process was used to assess the practitioners’ attitudes and perceived potential benefits of these apps for health care professionals. The domains covered were satisfaction of higher DMHA-based demand from new patients, improved patient satisfaction, time savings owing to efficiency gains, better quality of care for patients, greater treatment success, and additional treatment options.

Overall, the distribution of the answers represented a reasonably positive impression of practitioner-specific potential benefits, although with a slightly inferior portion of general agreement answers owing to conditioning by higher neutral and general disagreement responses.

The highest general agreement proportion of the answers was attributed to better time management owing to efficiency gains (112/160, 70%), closely followed by the benefit of having an additional treatment option (106/160, 66.3%). The lowest general agreement ratio was regarding the expectation of greater treatment success (62/160, 38.8%), which also demonstrated the highest neutral and second highest general disagreement shares.

For the general disagreement ratios, the highest proportion was described concerning the possibility of improving the quality of patient assistance (27/160, 16.9%), closely followed by the previously mentioned improved treatment success. The share of neutral answers ranged from 21.3% (34/160; regarding better time management owing to efficiency gains) to 46.3% (74/160; regarding improved treatment success).

Our adapted survey did not assess the perceptions on additional new patients, or additional income for this group of benefits, having added others regarding the satisfaction of higher DMHA-based demand and the DMHA as an additional treatment option at the suggestion of our survey reviewers.

#### Prescription Intentions of DMHAs

Of the 160 participants, 68 health care professionals (n=62, 91% psychologists; n=6, 9% psychiatrists) declared to have an increased likelihood (“Likely” or “Very Likely”) to prescribe DMHAs in the coming 12 months, representing 42.5% of all the answers.

There were some differences in the prescription intentions between the 2 professional groups analyzed. For this variable, psychologists revealed a 48% share of increased likelihood answers, whereas psychiatrists only had 22% of their responses corresponding to these.

Concerning professionals’ attitudes, 136 mental health care professionals (n=114, 84% psychologists; n=22, 16% psychiatrists) declared to have a generally positive (“Positive” or “Very Positive”) attitude toward the possibility of mental health professionals and psychologists being able to prescribe, recommend, or use clinically and technically validated DMHAs, representing 85% of all the answers. A much smaller difference was found in comparison with their intentions to prescribe. Psychologists and psychiatrists responded with 88% and 81%, respectively, generally positive attitude answers.

It is important to note while describing prescription intentions that practically no respondents to the survey have prescribed DMHAs to their patients. Simultaneously, there is no legally established prescription and reimbursement process in Portugal, as described in the *Introduction* section. This leads us to conclude that what we observe in this sample are the aprioristic perceptions and attitudes toward DMHAs.

Respondents who reported more positive attitudes toward DMHAs (*χ*^2^_1_=3.9; *P*=.048; Cramer V=0.19) and those who worked in a clinic (Fisher exact *P*=.03) reported a higher intention of prescription. Male respondents also reported a higher likelihood of assuming more positive attitudes toward DMHAs (Fisher exact *P*=.046). The demographic or work-related characteristics of other health care professionals were not significantly associated with either DMHA attitudes or prescription intentions.

We found a statistically significant association between the digital affinity score and prescription intention (Fisher exact *P*=.01). This was not the case for the association between the digital affinity and the mental health professionals’ attitudes (Fisher exact *P*=.67). Our results lead us to believe that Portuguese professionals expect to prescribe DMHAs shortly (the next 12 months); however, they are not currently very optimistic about these tools. This may suggest that they are open to changing their views.

#### Perceived Barriers to DMHA Prescription

Overall, 11 potential obstacles to DMHA prescription were listed. For most cases, except for lack of support from the manufacturer for technical issues, the answers demonstrated an agreement or total agreement with the characterization of the following as barriers to the prescription of these solutions.

With the highest share of agreement (154/160, 96.3%), the lack of information about digital apps gathered the most support from the respondents, including the highest percentage of “Totally Agree” answers. In addition, important issues such as the initial effort for health professionals (115/160, 71.9%), the need to adjust and adapt clinical records and practices (113/160, 70.6%), and ethical and legal questions (110/160, 68.8%) were very commonly identified as obstacles.

Respondents were found to disagree more frequently with the idea that uncertainty around informed consent (33/160, 20.6%) and data privacy and safety (28/160, 17.5%) would be substantial obstacles to DMHA adoption. At the same time, the most neutral answers (“Don’t Know” or “Neither Agree nor Disagree”) were registered regarding the lack of support from the manufacturer for technical issues (92/160, 57.5%), considerably superior to the next highest value (absence of copayment mechanisms; 56/160, 35%).

#### Measures to Support Adoption of DMHAs

In total, 10 different measures were presented to increase the adoption of DMHAs. For all cases, respondents expressed a favorable agreement with the utility of their adoption, ranging from 52% to 94% of the answers.

The highest share of agreement was reached concerning the need for more information about available DMHAs (151/160, 94.4%). Closely behind, most professionals considered that the existence of scientific evidence about the validity of the apps (147/160, 91.9%), the recommendation by professional and scientific societies (145/160, 90.6%), and the definition of the prescription process (138/160, 86.3%) along with the sharing and reporting of positive experiences by peers (137/160, 85.6%) were relevant measures to foster adoption.

The highest levels of disagreement were registered for the need to integrate DMHAs in health insurance plans (23/160, 14.4%), followed by changes to the legal framework (10/160, 6.3%) and manufacturer helplines for health care professionals (8/160, 5%). The most relevant share of neutral answers was registered regarding the integration of DMHAs in health insurance plans (54/160, 33.8%), which also reported the lowest level of agreement (83/160, 51.9%).

### KOL Analysis

#### Overview

Of the 25 contacted KOLs, 11 (44%) replied to our invitation to participate in the interviews, 7 (64%) of whom gave positive replies. One KOL did not show up for the scheduled interview, and the remaining 6 were interviewed during the period mentioned in the *Methods* section. The interviews started with an overview of the study by the coauthors. They proceeded to ask the KOLs what were, in their opinion, the 3 main benefits of, barriers to, and measures to support the adoption of DMHAs in the Portuguese context in descending order. An initial briefing was shared with the invitation to participate.

#### Perceived Potential Benefits From DMHAs and Attitudes Toward DMHAs

The 3 main benefits identified by the interviewed KOLs and that gathered consensus were the following:

- Improved access and accessibility to health care at the personal and population levels, including geographically more remote areas and preventive services.
- Improved efficiency in providing care, both from the point of view of direct cost (payment per treatment) and allocation of available human resources.
- Proximity to the user (including personalization of care, real-time monitoring, consideration of the user as an active participant, a more relevant number of potential users, and a potential for collecting real-world data for research).

#### Perceived Barriers to DMHA Prescription

The 3 main perceived barriers to DMHA adoption identified by interviewed KOLs and that gathered consensus were the following:

- Lack of knowledge and literacy of professionals at the digital skills level about existing DMHAs and related evidence-based information.
- Absence of training programs on DMHAs, especially those that positively position them and do not constitute them as a threat to professionals (especially as a risk of being replaced).
- The health system is not designed to consider digital tools (including their use, technical standards for information technology and information security, reimbursement, and stabilization of ethical and data protection concepts).

#### Measures to Support Adoption of DMHAs

The 3 main measures to support DMHA adoption identified by interviewed KOLs and that gathered consensus were the following:

- Creation of appropriate regulation, especially for clinical practice and reimbursement, and adequate health policies to boost the digital component safely.
- Promotion of literacy on mental health and training in digital apps (especially the younger generations), building awareness, and competency in digital tools in a constructive and collaborative perspective.
- Production of directives by the Ministry of Health and Professional Orders, such as an executive document with a selection of apps that could be useful and easy to implement while demonstrating good screening test characteristics.

#### Input Gathered From the KOLs

The second part of the interview consisted of the presentation of the results of the survey (as displayed in the previous subsections of the *Results* section for the web-based survey, ie, *Demographics*, *Perceived Potential Benefits From DMHAs and Attitudes Toward DMHAs*, *Prescription Intentions of DMHAs*, *Perceived Barriers to DMHA Prescription*, *Measures to Support Adoption of DMHAs*) to the KOLs and asking them whether they believed the obtained results to be aligned with their perception of most Portuguese psychiatrists and psychologists, justifying why.

Concerning the benefits of DMHAs, KOLs were aligned with the identified benefits for both users and professionals, as well as with the answer distribution of the sample. No KOL dissented from this view. KOLs expressed a perception of bias in the sample, reflecting a higher proportion of promoters of digital tools than the global average of mental health professionals. The answer to the hypothesis “improved ability to make informed choices” was the point that raised the most questions, and it is interesting to understand the rationale behind it. Greater adherence to treatment, access, disease management, and health literacy were in line with expectations. Response data were more balanced when evaluating the benefits for health professionals than for patients, where positive expectations seem to exist *a priori*.

Concerning the perceived barriers to the adoption of DMHAs, KOLs were generally aligned with identified benefits and the distribution of the answers in the survey, with only 1 KOL stating that they did not think these were representative of their peers’ opinions. KOLs considered that respondents had a favorable perception of digital apps, with a lack of technical support from the manufacturer and the need to adjust work processes surprising them. The first item was a surprise owing to the low degree of disagreement; the second item surprised them because it entailed that the adaptation process would necessarily be painful. In the KOLs’ opinion, professionals need help formulating the problems that concern them the most and whether or not it is a problem. They attributed these issues to a lack of experience with DMHAs. Furthermore, KOLs stated that they expected that a higher percentage would agree with the lack of reimbursement owing to being included in insurers’ commercial packages as a relevant barrier.

Finally, regarding the measures to support the adoption of DMHAs, the KOLs aligned with the adoption support measures identified in the survey and the distribution of responses. The action “integration of applications in health insurance” generated the most comments. KOLs considered that it could reflect ≥1 of the following 3 issues: *a priori* concerns about data privacy (including data sharing with third parties), low payment fees to professionals, or matters related to stigma.

In addition, KOLs pointed out several peculiarities of the Portuguese context that they believed were important for any stakeholder (government, business, academia, or others) who wishes to develop a likely successful DMHA to address mental health professionals’ needs. These were as follows:

- Health system financing and the incentives it produces must be considered, as health systems with budgeting practices based on production estimates instead of outcomes will experience severe difficulties in monetizing DMHAs and validating them as productive or cost-effective investments.
- Even if they are not inferior to other interventions, DMHAs may allocate resources more effectively and deliver savings by shifting individuals with lower mental health care needs to DMHAs and allowing mental health professionals to focus more of their time and attention on more complex cases.
- Professionals’ resistance to novelty and workflow change, as well as negativity bias and feelings of being replaced by apps, must be addressed to ensure a successful embracement of DMHAs. KOLs considered this to be particularly true for psychiatrists, supported by the number of people who answered that they had the expectation of increased initial effort for health professionals (72% of the survey’s respondents), need to adjust and adapt clinical records (71%), and additional workload (56%).
- DMHAs must be adapted for use in a clinical setting, namely for severe mental issues (such as schizophrenia), where DMHAs are currently unfit to deal with acute severe episodes. Furthermore, DMHAs must be balanced to prevent a user’s perception of pseudoautonomy that leads to the early abandonment of therapeutic interventions. Further research and development are required in these areas.
- Stigma plays a key role. This is not exclusive to one type of actor and ranges from the perceptions that professionals have of users and patients to insurance companies’ pricing policies and offers. Information sharing with third parties other than professionals and users must be selective and scrutinized to prevent distrust in these tools and to avoid discrimination toward people who use them.
- Any DMHA must bear in mind cybersecurity risks and their impact on the user. Mental health issues are usually intimate matters, and that places a higher emphasis on information security.
- Research and development on DMHAs must be ongoing, both from a clinical and a technical standpoint. Evidence generation, treatment, and analysis are expected to be performed on a rolling basis owing to their digital nature.

## Discussion

### Principal Findings

#### Perceived Benefits, Barriers, and Measures to Foster Adoption

Regarding the perceived benefits of DMHAs for patients, our findings were generally aligned with those of the study by Dahlhausen et al [55] despite notable differences. Portuguese respondents were less optimistic about improved access to care and more positive about enhanced adherence to treatment. The time-saving potential benefit generated the most positive responses, whereas it was the worst regarding the perceptions of shared questions in the study by Dahlhausen et al [55]. The same is true for patient satisfaction, which was almost evenly split in the study by Dahlhausen et al [55]. Conversely, Portuguese professionals had some of the least positive perceptions concerning treatment success and quality of patient assistance. In contrast, German professionals ranked some of their most positive scores for this variable. Overall, participants demonstrated a very high agreement with the listed potential benefits.

The respondents were less enthusiastic about the perceived benefits of DMHAs for professionals, namely, regarding the expectation of improved treatment success and the possibility of improving the quality of patient care. They did recognize the benefits of better time management owing to efficiency gains and the benefit of having additional treatment options.

We found a wider gap between the practitioners’ attitudes toward DMHAs and their intentions to prescribe them in comparison with German professionals. This may be because of the effects of social desirability bias on provided answers [63-66] and the consequent positive aprioristic expectations. Their role might be expanded in the provided answers given the absence of a regulatory track for prescription and payment and general knowledge about DMHAs.

Concerning the barriers to DMHA adoption, our respondents agreed more with the importance of the lack of information about digital apps and the initial effort for health professionals, as well as the need to adjust and adapt clinical records and practices, alongside ethical and legal questions. They demonstrated general neutrality toward the importance of the lack of support from the manufacturer for technical issues and reimbursement schemes, and KOLs have attributed this neutrality to a lack of knowledge about these tools or concerns with patient data privacy.

By contrast, these mental health professionals agreed that more information about DMHAs, increased scientific evidence about their validity, recommendations by professional and scientific societies, and the definition of a prescription process along with the sharing and reporting of positive experiences by peers were all relevant measures to foster adoption.

To leverage DMHA adoption, both Portuguese and German professionals recognized the importance of the first 2 points and concurred on classifying direct exchange with developers as one of the least important issues. They disagreed, however, on the necessity of integrating apps into health insurers’ commercial packages.

#### Attitudes and Prescription or Recommendation Intentions

In the German study, health care professionals with higher digital affinity were considerably more positive toward attitudes and prescription intentions; however, the strength of the associations was weak. While DiGA is already at work, DMHAs do not currently have a clear path for partaking in Portugal’s clinical process and care provision.

Although a direct comparison between the professional groups of this study and the ones in the replicated paper is not immediate, the strictest association occurs in practitioners in psychiatric specialties (“child and adolescent psychiatry and psychotherapy” and “psychiatry and psychotherapy”), corresponding to the Portuguese psychiatrists, and the remaining specialties (“psychological psychotherapy” and “psychosomatic medicine and psychotherapy”), corresponding to psychologists in the Portuguese case.

The first group’s differences between attitude and intention toward recommendation or prescription ranged from 38.5% to 45.9%, whereas the second group’s differences ranged from 35.2% to 36.6%. In our study, for psychiatrists, we found a difference of 59% between the reported positive attitudes toward DMHAs and their intention to prescribe them (between 81% and 22%, respectively). For psychologists, the same difference was 40% (between 88% and 48%, respectively).

Although Portuguese psychologists’ answers are more favorable than their German counterparts, the differences between attitudes and intentions have a similar magnitude. However, more Portuguese psychiatrists presented positive attitudes, while slightly fewer reported prescription intentions, thus yielding a larger difference than the one found in German psychiatrists.

#### KOL Analysis

First, it is important to highlight that in a country with no payment or reimbursement tracks or clinical or technical validation standards specific to digital health apps, these findings are based on the individual KOL’s experience and perception of the national landscape.

Second, the interviewed KOLs mainly considered that the survey’s results, despite the sample size and a possible bias in favor of digital tools, were representative of the study’s intended population. This increased our confidence in the obtained results and, consequently, in the conclusions they can draw toward DMHA promotion.

Third, the fact that KOLs were unanimous regarding several issues—such as access to care, patient centricity, or (need for) mental health professionals’ digital literacy draws attention to the fact that the Portuguese health system needs profound transformation. Alerts have been abundant since Europe’s health systems suffered from the COVID-19 pandemic and its clinical backlog [67], which have come on top of long-lasting struggles such as workforce skills, motivation, and retention [68,69]; speed of digital transformation [70]; or the need to foster innovation [71]. The reference to these topics in unison between knowledgeable people, in a blinded and independent fashion, strengthens these arguments and reinforces the need to act on a broader digital health strategy that encompasses digital health applications.

### Relationship With the Wider Portuguese Health System

The reported results from the survey and the KOL analysis revealed a general immaturity in implementing DMHAs (digital medical products, services, and interventions in general) in Portugal.

The survey shows an explicit generalized agreement with the perceptions listed concerning potential benefits, barriers, or measures to foster adoption. These results seem polarized to one of the extremes, as disagreement answers were never >21% for the specific questions.

Moreover, the topics generating the most neutral answers require some practical implementation of these tools—specifically, on the improvement of patient satisfaction, treatment success, and capability to make informed choices; on the lack of support from the manufacturer for technical issues; and on reimbursement of medical prescription, as well as integration of DMHAs in health insurance coverage. We hypothesize that this neutrality confirms a lack of practical experience with these tools. Otherwise, professionals would have more positive or negative perceptions because of their experience and less ambiguity or one-way polarization.

Our theory is compatible with other findings from the survey; although Portuguese professionals are at least as positive in their attitudes toward DMHAs as German professionals, the former group has only a smaller share of those who do intend to prescribe them, thus generating a wider gap between attitudes and prescription intentions for Portuguese mental health professionals than that found for German counterparts in the study by Dahlhausen et al [55]. If their intentions to prescribe are inferior to their German counterparts, then it is expected that they will do so less often, aggravating the know- difference in the practical knowledge of using these tools. They also differ regarding the perceived importance of required workflow adjustments as a limitation to adoption.

This theory is also compatible with the Portuguese health system paradigm concerning mental health as described by the KOLs, who repeatedly reported the professionals’ resistance to change and novelty, as well as the intrinsic fear of being replaced by DMHAs, as barriers to DMHA adoption. This barrier should be taken seriously so as not to create a negative reinforcement loop that further restrains professionals from adopting and applying such tools.

This is all the truer as the Portuguese National Health Plan [72] ranks “access to mental healthcare” as the sixth most important health determinant for the country’s needs but fails to set any of its 37 health objectives to address this issue and its consequences. A specific National Program for Mental Health, responsible for producing a National Plan for Mental Health, existed from 2008 [73] until 2020. The National Coordination for Mental Health Policies replaced it at the beginning of 2022 [74]. Although the former has considerably failed to enact mental health care reform [75] and had no mention of DMHAs, the new Coordination is yet to publish its plan and objectives.

### Strengths and Limitations

Portugal faces important challenges despite being a relatively small country (approximately 10.5 million inhabitants), namely, a rapidly growing aging population [76], declining birth rates [77], and an overburdened health system [46]. These challenges are similar to those encountered in many high-income countries. Therefore, Portugal might serve as a test bed to validate digital solutions that ease the workload on health care providers and increase patient autonomy. Our study contributes to this understanding as it is, to the best of the authors’ knowledge, the first work of its kind on the Portuguese landscape concerning DMHAs.

The study elicits preferences and issues that are clearly important to understand the demand side, as it is visible, for example, through the high degree of agreement in survey answers and KOL responses. Furthermore, the mixed methods methodology allows us to combine the perspective of those closest to potential users with a helicopter view with in-depth knowledge, making the derived conclusions more robust.

All biases inherent to sampling and KOL selection are potential limitations of this study, with the social desirability of the provided answers and the role of expectation in the survey’s answer process (ie, the belief respondents might have that they are expected to answer more favorably about technology than they would otherwise do), as well as self-selection, being among the most relevant. Their impact on provided answers is mitigated by the fact that in a postpandemic reality, telehealth (albeit limited to teleconsultation volume) [53] has proven its benefits to a large extent and certainly more than at the time Dahlhausen et al [55] conducted their study.

The sample number may constitute a further limitation, as 160 answers only partially characterize a population of 1528 psychiatrists and an undetermined number of psychologists. However, the level of agreement between the elements gathered in the survey and the KOLs’ assessment, both before and after seeing the survey results, leads us to believe that the underlying uncertainty in the provided answers might not be as considerable as expected. Furthermore, the proportion between psychologists and psychiatrists in our study and the study by Dahlhausen et al [55] is approximately the same (6 to 1, respectively). This is a relevant sample characteristic that favors the comparison between the results of both studies.

Finally, the comparability between studies is limited by their different scopes and stages of regulatory development: the study by Dahlhausen et al [55] was produced when DiGA was starting to roll out and for all digital health applications (ie, not exclusive to mental health); as of the conclusion of this study, Portugal does not have a payment or reimbursement system in place for digital health apps in general, or DMHAs specifically. Even then, the similarity of methods, presentation of results, and the produced discussion allow, in our view, for a proper comparison.

### Future Work

From a general perspective of digital health apps, it would be useful to fully replicate this study to understand which points are common to all apps and which are solely applicable to DMHAs. Deloitte’s report for Health Cluster Portugal [78] is the only known work on the Portuguese domestic market. However, its high-level nature reveals how immature the market is in terms of digital health apps. More research is needed to understand their market dynamics, namely, when it comes to the expectations of supply and demand sides. To further improve the interpretation of results and have a clearer sense of the actual differences between Portuguese mental health professionals and their German counterparts, it would be helpful to implement this or similar surveys on the overall Portuguese population. By evaluating the differences between mental health professionals and the people they serve, one could distinguish between context and specific differences attributable to training or skillset.

Moreover, as this is a portrait of the landscape as far as DMHA are concerned and one of the main difficulties that we felt was survey engagement (despite extensive dissemination efforts), it would be essential to perform this extended work with further engagement from professional orders, professional societies, and market-based stakeholders (eg, developers of apps such as 29kFJN [79]). Outreach to international stakeholders such as BfArM, mobile health Belgium, and market operators with products that have already secured regulatory approval by them would be beneficial.

Given that this necessarily entails regulation, it would be interesting to expand further on the regulatory science angle and the opportunity for either regulatory tracks at the EU level (following calls for European Health Union and increasing competencies of the EU in health care [80]) or regulatory replication to ensure a fair, competitive, and innovative digital health market in the EU. This is in the interests of regulators, companies, and citizens.

Finally, it is widely agreed that digital interventions have appreciable potential to deliver more and better health care. However, if these—or any other health interventions—are to succeed, they need rigorous planning by diagnosing the status, defining targets and priorities, establishing objectives and desirable results, and outlining the best evidence-based strategies and plans to achieve them [81]. Monitoring and evaluating the attained results and deriving learned lessons are also necessary. In the authors’ view, this road map for DMHAs would potentially be the most relevant future work that could be done.

### Conclusions

Portuguese mental health professionals’ perceptions of digital health apps present clear aprioristic expectations regarding the benefits of DMHAs for users, especially concerning improved therapeutic adherence and health literacy. Although professionals generally recognize the benefits for patients, they are less optimistic about the expected advantages for themselves and their peers. Although the usefulness of DMHAs for professionals needs to be clarified, benefits such as efficiency gains and having an additional treatment option are among the most valued benefits from the onset.

Chief among the main perceived barriers are the need for more information about digital health apps, preconceptions of initial use efforts for health professionals, and the need to adjust and adapt clinical records. The main enablers of DMHA use identified include more information about these apps, both regarding how they work and scientific evidence about the validity of such apps, as well as recommendations by professional and scientific societies. Governmental or regulatory guidelines are strongly recommended.

Portuguese mental health professionals, compared with German mental health professionals, were similar in most of the reported answers. Some notable differences were fewer positive perceptions concerning treatment success and quality of patient assistance, a wider gap between attitudes and prescription intentions for Portuguese mental health professionals, and the need for considerable workflow adjustments as limitations for adoption.

Concerning how digitally literate mental health professionals perceive themselves and their patients to be, the scores of digital literacy–related issues in terms of barriers and measures to support adoption in the survey, along with the conclusion by the KOLs that this is one of the main issues faced by mental health professionals in Portugal, lead us to posit that mental health professionals perceive themselves to have high degrees of digital illiteracy. They also perceived a strong need for patients to be educated should DMHAs or other digital tools be implemented to deliver mental health care.

Mental health professionals believe that their role in digitalizing health care provision consists mainly of promoting literacy among peers, namely, to and by younger age groups, thus forming communities able to capacitate a growing number of professionals. Their participation in professional and scientific societies is another avenue for further engagement. Finally, mental health professionals believe that the Portuguese government sector should play a crucial role in shaping the health system and enabling the proper organizational and financial means and incentives to catalyze transformation.

## Appendix

Multimedia Appendix 1 Translated adapted survey questionnaire (Portuguese to English).

Multimedia Appendix 2 Translated original survey questionnaire (English to Portuguese).

Multimedia Appendix 3 Summary of modifications between survey questionnaires.

## Abbreviations

CHERRIES: Checklist for Reporting Results of Internet E-Survey
DiGA: Digitale Gesundheitsanwendungen
DMHA: digital mental health app
EU: European Union
KOL: key opinion leader

## Data Availability

The data sets generated or analyzed during this study are not publicly available but are available from the corresponding author upon reasonable request.
