# Supplementary material for: Mental Health Professionals’ Attitudes Toward Digital Mental Health Apps and Implications for Adoption in Portugal: Mixed Methods Study
Source: JMIR Hum Factors. 2023 Jun 2;10:e45949. doi: 10.2196/45949 (PMC10276319; doi:10.2196/45949)
Supplement: Multimedia Appendix 1 [file humanfactors_v10i1e45949_app1.docx]

MULTIMEDIA APPENDIX 1

Survey - Mental Health Professionals

*What are mental health professionals' perceptions of digital health apps? How digitally informed do these professionals consider themselves to be, and what are their perceptions of how digitally literate are their patients/users/clients? How do mental health professionals anticipate their own role in the digital transition of healthcare, and what do they see as the greatest benefits, risks, challenges and opportunities?*

Within the framework of a research carried out in the PhD Programme in Health Data Science at the Faculty of Medicine of the University of Porto, this study investigates the attitudes of psychiatrists and psychologists towards digital health apps, including those who have had little or no previous contact with digital health apps. The goal is to help professionals deal with this new screening, diagnostic, and therapeutic approach, as well as to determine the level of knowledge of mental health professionals. Completing the survey will take 4-7 minutes.

*What are digital health apps?*

Digital health apps were designed to help track, monitor, diagnose, and treat or minimise signs/symptoms of a given disease/disorder.

*Is there a regulatory framework for digital health apps?*

In Portugal, digital health apps are subject to the CE marking and, in some cases, to the Medical Devices Regulation, and it is not possible to validate them for access to health technologies such as medicines.

The DiGA programme (Digitale Gesundheitsanwendungen, or "digital health apps" in German) was implemented in Germany in October 2020 in order to define a clear procedure that enables digital health apps to be approved as medical devices and additionally licensed by the Federal Institute for Medicines and Medical Devices (BfArM). DiGA apps can be prescribed by doctors and are reimbursed by all public health services - as such they are also called "medical prescription apps". As of July 1^st^, 2022, 34 apps had received regulatory approval through this process, and 14 of these were considered to respond to psychological/psychiatric pathologies. The success of this programme to this date has triggered interest from French health authorities, who aim to develop a similar process by the end of 2022.

In Belgium, the mHealthBelgium programme started making assessments and reimbursement recommendations in January 2021, after being announced in 2018. Based on an assessment consisting of a pyramid representing three stages of validation (legal and regulatory issues, secure communication and privacy, and financing and reimbursement), this programme had 36 digital health apps with some level of validation as of June 2^nd^, 2022.

We therefore ask you to complete this survey drawing mainly from your own experience. In order to guarantee maximum comparability between Portugal and Germany (where the use of validated digital health apps is reimbursable) in this context, we ask you to answer the following questions **assuming digital health apps meet all regulatory requirements and are safe, of reliable quality, and effective**.

This survey is being conducted as part of a PhD thesis for the PhD Programme in Health Data Science at the FMUP. The person responsible for collecting data is the student Diogo Nogueira Leite (202002508), who can be contacted at up202002508@up.pt for further questions.

This survey is comprised of 27 question(s).

# Attitudes and perceptions about digital health apps


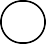


[Q1]

Please indicate your level of agreement with the following sentence:

“*I am in favour of allowing doctors and psychologists to prescribe/recommend/use technically validated clinical digital health apps."* *

Please select **only one** of the following options:


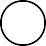
 Strongly disagree

Disagree


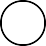
 Neither agree nor disagree
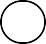
 Agree


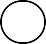
 Strongly agree


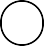

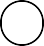


[Q3]

How would you rate your ability when it comes to... *

Please select the appropriate position for each element:


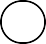


[Q2]

Please indicate your level of agreement with the following sentence:

*"I feel sufficiently informed about digital health apps to recommend or inform about them."* *

Please select **only one** of the following options:

Strongly disagree
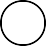
 Disagree


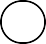
 Neither agree nor disagree


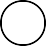
 Agree


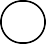
 Strongly agree

|  | **Very**  **bad** | **Bad** | **Neither good**  **nor bad** | **Good** | **Very**  **good** |
| --- | --- | --- | --- | --- | --- |
| **...analyse the range of digital health applications available?** | 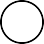 | 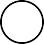 | 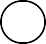 | 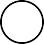 | 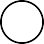 |
| **...giving my patients/users/clients/**  **people who consult me information about digital health apps?** |  |  | 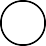 | 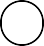 | 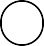 |
| **...distinguish between good and bad digital health apps?** | 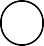 | 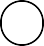 | 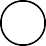 | 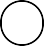 | 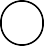 |


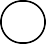


[Q4] How regularly do your patients/users/clients ask you about digital health apps in general or about your prescription? *

Please select **only one** of the following options:

Never


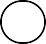
 Less than once a month
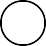
 On a monthly basis


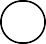
 On a weekly basis
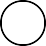
 On a daily basis


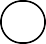


[Q5]

Please indicate your level of agreement with the following sentence:

“*My patients/users/clients expect that I will prescribe/advise digital health apps." **

Please select **only one** of the following options:


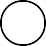
 Strongly disagree

Disagree


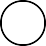
 Neither agree nor disagree
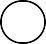
 Agree


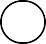
 Strongly agree


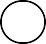


[Q6]

How regularly do you give information about digital health applications? *

Please select **only one** of the following options:


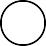
 Never


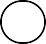
 Less than once a month
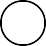
 On a monthly basis


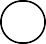
 On a weekly basis
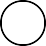
 On a daily basis


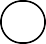


[Q7]

How likely are you to recommend digital health apps in the next 12 months? *

Please select **only one** of the following options:

Very unlikely
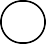
 Unlikely


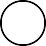
 Likely


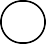
 Very likely
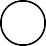
 No opinion


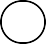


[Q8]

Please indicate your level of agreement with the following sentence:

“*I am more likely to recommend digital health apps to young patients/users/clients."* *

Please select **only one** of the following options:

Strongly disagree
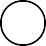
 Disagree


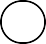
 Neither agree nor disagree
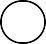
 Agree


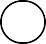
 Strongly agree


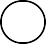


[Q9]

Please indicate your level of agreement with the following sentence:

“*I have sufficient knowledge of cybersecurity, processing of personal data, and other technical elements associated with my current activity."* *

Please select **only one** of the following options:

I have no knowledge of this kind
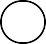
 Very low


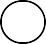
 Low


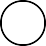
 Medium
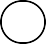
 High


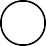
 Very high

## [Q10]

### In your opinion, what are the main benefits of digital health apps for patients/users/clients? *

Please select the appropriate position for each element:

|  | **Strongly disagree** | **Disagree** | **Neither agree nor disagree** | **Agree** | **Strongly agree** | **I don’t know** |
| --- | --- | --- | --- | --- | --- | --- |
| **Improved ability of patients/ /users/clients to make informed choices, e.g. additional treatment options or treatment according to clinical guidelines** | 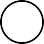 | 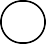 | 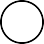 | 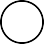 | 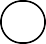 | 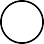 |
| **Proper disease management, e.g. increased coordination between all those involved in treatment processes or better management of disease-related difficulties in daily life** | 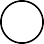 | 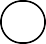 | 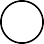 | 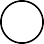 | 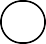 | 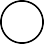 |
| **Wider adherence to treatment e.g. regular reminders about agreed treatment and motivation to adopt healthy behaviours** | 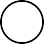 | 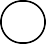 | 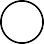 | 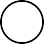 | 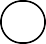 | 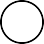 |

|  | **Strongly disagree** | **Disagree** | **Neither agree nor disagree** | **Agree** | **Strongly agree** | **I don’t know** |
| --- | --- | --- | --- | --- | --- | --- |
| **Improved access to healthcare, e.g. bridging the gap between waiting times for treatment, offering more comprehensive care or minimum levels of care** | 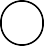 | 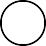 | 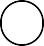 | 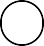 | 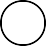 | 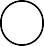 |
| **Increased health literacy, e.g. through better education of the population** | 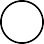 | 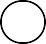 | 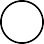 | 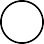 | 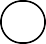 | 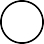 |

[Q10b]

If you think there are other benefits of digital health apps for patients/users/clients that have not been listed in the previous table, please use the space below to mention which ones (optional).

Please write your answer here:

## [Q11]

### In your opinion, what are the main advantages of digital health apps for healthcare professionals? *

Please select the appropriate position for each element:

|  | **Strongly disagree** | **Disagree** | **Neither agree nor disagree** | **Agree** | **Strongly agree** | **I don’t know** |
| --- | --- | --- | --- | --- | --- | --- |
| **Satisfaction resulting from a greater demand from new patients/users/clients** | 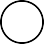 | 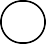 | 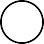 | 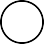 | 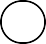 | 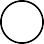 |
| **Increased patient/user/client satisfaction** | 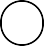 | 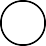 | 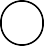 | 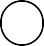 | 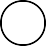 | 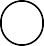 |
| **Time savings per patient/user/client due to efficiency gains** | 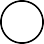 |  |  |  |  |  |
| **Better quality of care for the patient/**  **user/client** |  |  |  |  |  |  |
| **Greater treatment success** |  |  |  |  |  |  |
| **Additional treatment option** |  |  |  |  |  |  |

[Q11b] If you think there are other benefits of digital health apps for healthcare professionals that were not listed in the previous table, please use the space below to mention which ones (optional).

Please write your answer here:

## [Q12]

### What do you consider to be the biggest challenges/obstacles to the recommendation and/or use of digital health apps? *

Please select the appropriate position for each element:

|  | **Strongly disagree** | **Disagree** | **Neither agree nor disagree** | **Agree** | **Strongly agree** | **I don’t know** |
| --- | --- | --- | --- | --- | --- | --- |
| **Lack of information on digital health apps, e.g. on available apps and their suitability in the clinical setting** |  |  |  |  |  |  |
| **Lack of scientific evidence or insufficient scientific evidence on the benefits for patients** |  |  |  |  |  |  |
| **Level of initial training and/or familiarisation effort for health professionals** |  |  |  |  |  |  |
| **Need for adjustments and adaptations of existing intervention and/or treatment processes and/or clinical practice** |  |  |  |  |  |  |

|  | **Strongly disagree** | **Disagree** | **Neither agree nor disagree** | **Agree** | **Strongly agree** | **I don’t know** |
| --- | --- | --- | --- | --- | --- | --- |
| **Poor integration or compatibility with existing software and clinical practice tools** |  |  |  |  |  |  |
| **Lack of support from the manufacturer on problems or technical issues** |  |  |  |  |  |  |
| **Permanent increase in the workload of doctors and psychologists due to additional tasks in patient care** |  |  |  |  |  |  |
| **Absence of definition on co-payment mechanisms for the use of digital health apps or accompanying medical/therapeutic services required by them** |  |  |  |  |  |  |
| **Uncertainty about privacy and security of personal (health) data** |  |  |  |  |  |  |

|  | **Strongly disagree** | **Disagree** | **Neither agree nor disagree** | **Agree** | **Strongly agree** | **I don’t know** |
| --- | --- | --- | --- | --- | --- | --- |
| **Uncertainty about informed consent issues (e.g. ability to withdraw consent)** |  |  |  |  |  |  |
| **Other ethical issues and legal ambiguities,**  **e.g. in relation to the risk of liability in case of incorrect diagnosis or treatment based on data from the app** |  |  |  |  |  |  |

[Q12b] If you think there are other challenges/obstacles to the recommendation and/or use of digital health apps for patients/users/clients that were listed in the previous table, please use the space below to mention which ones (optional)

Please write your answer here:

## [Q13]

### What might encourage you to recommend and/or use digital health apps? *

Please select the appropriate position for each element:

|  | **Strongly disagree** | **Disagree** | **Neither agree nor disagree** | **Agree** | **Strongly agree** | **I don’t know** |
| --- | --- | --- | --- | --- | --- | --- |
| **More information on the available apps and their suitability for the underlying health objective (screening, treatment, description by pathology, etc.)** |  |  |  |  |  |  |
| **Definition of prescribing process / advice and use guidelines** |  |  |  |  |  |  |
| **Legislative framework change on screening, diagnosis, treatment and prognosis options that may be recommended and/or used by healthcare professionals** |  |  |  |  |  |  |
| **Existence of scientific evidence of the validity of the apps as a health intervention** |  |  |  |  |  |  |

|  | **Strongly disagree** | **Disagree** | **Neither agree nor disagree** | **Agree** | **Strongly agree** | **I don’t know** |
| --- | --- | --- | --- | --- | --- | --- |
| **Recommendation of specific apps by health institutions (DGS, INFARMED, EMA, etc.)** |  |  |  |  |  |  |
| **Integration of apps as part of the commercial packages provided by health insurers** |  |  |  |  |  |  |
| **Issues raised or requests made by patients/clients/**  **users themselves** |  |  |  |  |  |  |
| **Helpline provided by the manufacturer/**  **developer of a digital health app** |  |  |  |  |  |  |
| **Reporting/sharing positive experiences of colleagues, e.g. at conferences or in peer-reviewed journals** |  |  |  |  |  |  |
| **Recommendation of one or more apps by specific clinical guidelines/**  **professional societies** |  |  |  |  |  |  |

[Q13b] If you think there are other factors that might encourage you to prescribe digital health apps that were not listed in the previous table, please use the space below to mention which ones (optional)

Please write your answer here:

### [Q14] Please indicate which information on digital health apps you would be interested in if it were possible, in the Portuguese context, to prescribe/recommend them? *

Please select **all** that apply:

What apps are there?

For which pathologies or other indications is an app suitable? What are the proven benefits and risks of an app?

How much does a digital health app cost? Does the National Health Service and/or health insurance companies pay for it? In what way?

How do I prescribe/recommend the use of an app?

How do my patients/users/clients get an app after I prescribe/recommend it, and how should they use it?

How do I monitor the use of an app?

I do not wish to receive any further information about apps.

Other:

[Q15] Please further develop your views on digital health apps (optional).

Please write your answer here:

# Sociodemographic questions

[Q01]

Which of these describes your occupation? *

Please select **only one** of the following options:

Psychologist

Child and adolescent psychiatrist Adult psychiatrist

Other

[Q02] You work in a geodemographic area with *

Please select **only one** of the following options:

More than 500,000 inhabitants

100,001 – 500,000 inhabitants

20,001 – 100,000 inhabitants

5,001 – 20,000 inhabitants Less than 5,000 inhabitants

[Q03]

Which type of workplace best describes your current occupational status? (select all that apply) *

Please select **all** that apply:

Hospital

Primary health care Clinic

Private practice (no other colleagues)

Practice with other colleagues (e.g., joint or group practice)

Other:

[Q04]

Do you work in the National Health Service (SNS), in the private sector, or in both? *

Please select **only one** of the following options:

Only in SNS

Only in the private sector In both

[Q05] How many doctors or psychologists (cf. the case) work at your workplace?

Please write your answer here:

[Q06]

What is your level of digital interaction in a professional context, on a scale of 1 (no digital interaction at all) to 10 (a lot of digital interaction)? *

 The answer must be between 1 and 10

 You can only enter a whole number in this field.

Please write your answer here:

[Q07] What is your age group? *

Please select **only one** of the following options:

Under 26 years old 26 – 35 years old

36 – 45 years old

46 – 55 years old

56 – 65 years old Over 65 years old

[Q08]

What gender do you identify as? *

Please select **only one** of the following options:

Female Male

Other

Should you wish to be contacted to receive a summary of the results arising from this survey in a published study, please complete this form (https://inqueritos.up.pt/index.php?r=survey/index&sid=489829&lang=pt).

Thank you for your participation and contribution to the research!

Thank you for completing this survey.
