# Supplementary material for: Mental Health Professionals’ Attitudes Toward Digital Mental Health Apps and Implications for Adoption in Portugal: Mixed Methods Study
Source: JMIR Hum Factors. 2023 Jun 2;10:e45949. doi: 10.2196/45949 (PMC10276319; doi:10.2196/45949)
Supplement: Multimedia Appendix 2 [file humanfactors_v10i1e45949_app2.docx]

# Questionário de investigação para profissionais de saúde

*(O questionário foi apresentado a inquiridos na Alemanha. Esta cópia foi automaticamente traduzida, apresentando apenas as questões incluídas na publicação.)*

# Página introdutória

*Que aplicações DiGA estão disponíveis? Como é que funcionam? Que benefícios oferecem e a quem? Como é que prescrevo uma? Como é que os meus pacientes a obtêm e utilizam, e como é que posso apoiá-los?*

As respostas a estas e outras questões podem ser encontradas na pasta de informação “DiGA for your practice: What I need to know now”, que pode descarregar sem qualquer custo depois de preencher o seguinte questionário da Universidade de Witten/Herdecke. De uma forma breve e clara, a pasta contém as informações mais importantes sobre as aplicações DiGA para a sua prática clínica.

No âmbito de uma investigação de doutoramento na Faculdade de Saúde, este estudo investiga as atitudes de psicoterapeutas e médicos de todas as especialidades em relação às aplicações digitais de saúde (DiGA), incluindo aqueles que tenham tido pouco ou nenhum contacto prévio com aplicações digitais de saúde. O objetivo é ajudar os profissionais a lidar com esta nova forma de assistência. O preenchimento do questionário demorará 4-7 minutos.

Obrigado pela sua participação e contribuição para a investigação!

# Consentimento do participante

Este questionário é anónimo e não revelará a sua identidade. Todos os dados do questionário serão usados unicamente para fins de investigação científica. A participação é voluntária. Antes de avançar, pedimos-lhe que leia a informação sobre o tratamento de dados no contexto deste estudo.

Ao clicar no botão abaixo, reconhece que leu e consente com a informação em anexo. Li e concordo com a informação sobre o tratamento dos dados do questionário.

# Página informativa: Em que é que as aplicações DiGA diferem de outras aplicações de saúde?

*O que são as DiGA?*

As DiGA são aplicações digitais para pacientes que ajudam a detetar, monitorizar e tratar ou aliviar a doença.

*O que distingue as aplicações DiGA de outras aplicações de saúde?*

As aplicações DiGA são aprovadas como dispositivos médicos e adicionalmente licenciadas pelo Instituto Federal de Medicamentos e Dispositivos Médicos (BfArM). As aplicações DiGA podem ser prescritas por médicos e psicoterapeutas e são comparticipadas por todos os serviços de saúde públicos – como tal, também são denominadas “aplicações com receita médica”.

*Que aplicações DiGA estão disponíveis?*

Até à data, há 6 aplicações DiGA aprovadas: para o tratamento do zumbido (“kalmeda”), transtornos de ansiedade (“velibra” e “invirto”) e obesidade (“zanadio”), bem como a “somnio” para o treino do sono e a “vivira” para terapia com exercício. Há várias outras aplicações numa vasta gama de indicações que estão atualmente no processo de aprovação.

Pedimos-lhe que preencha este questionário unicamente a respeito das aplicações DiGA (“aplicações com receita médica”) e não das aplicações de saúde em geral.

# Perguntas do questionário sobre as aplicações DiGA

Qual o seu nível de concordância com a frase seguinte? “Sou a favor da possibilidade de médicos e terapeutas prescreverem aplicações digitais de saúde (“aplicações com receita médica”).”

o Discordo totalmente

o Discordo o Não consigo decidir o Concordo

o Concordo totalmente

Qual o seu nível de concordância com a frase seguinte? “Sinto-me suficientemente informado sobre as aplicações digitais de saúde (“aplicações com receita médica”) para as prescrever.”

o Discordo totalmente

o Discordo o Não consigo decidir o Concordo

o Concordo totalmente

Como classifica a sua competência no que toca a...

| Muito má | | Má | Nem boa nem má | Boa | Muito boa |
| --- | --- | --- | --- | --- | --- |
| ...analisar o conjunto de aplicações digitais de saúde disponíveis? | o | o | o | o | o |
| ...aconselhar os pacientes sobre aplicações digitais de saúde? | o | o | o | o | o |
| ...distinguir entre as boas e as más aplicações digitais de saúde? | o | o | o | o | o |

Com que regularidade é que os seus pacientes o questionam sobre aplicações digitais de saúde (“aplicações com receita médica”) em geral ou a propósito da sua prescrição?

o Nunca

o Menos do que uma vez por mês

o Mensalmente o Semanalmente o Diariamente

Qual o seu nível de concordância com a frase seguinte? “Os meus pacientes esperam que eu prescreva aplicações digitais de saúde (“aplicações com receita médica”).”

o Discordo totalmente

o Discordo o Não consigo decidir o Concordo

o Concordo totalmente

Com que regularidade prescreve aplicações digitais de saúde (“aplicações com receita médica”)?

o Nunca

o Menos do que uma vez por mês

o Mensalmente o Semanalmente o Diariamente

Qual a probabilidade de prescrever aplicações digitais de saúde (“aplicações com receita médica”) nos próximos 12 meses?

o Muito improvável o Improvável o Não consigo decidir o Provável o Muito provável

Qual o seu nível de concordância com a frase seguinte? “Tenho maior probabilidade de prescrever aplicações digitais de saúde (“aplicações com receita médica”) a pacientes jovens.”

o Discordo totalmente

o Discordo o Não consigo decidir o Concordo

o Concordo totalmente

Na sua opinião, quais os principais benefícios das aplicações digitais de saúde (“aplicações com receita médica”) para os pacientes?

| Discordo totalmente | | Discordo | Não consigo decidir | Concordo | Concordo totalmente | Não sei |
| --- | --- | --- | --- | --- | --- | --- |
| Benefícios diretos de saúde, p. ex., opções adicionais de tratamento ou tratamentos mais à base de diretrizes | o | o | o | o | o | o |
| Melhor gestão da doença, p. ex., maior coordenação dos processos de tratamento ou melhor gestão das dificuldades relacionadas com a doença no dia a dia | o | o | o | o | o | o |
| Maior adesão ao tratamento, p. ex., lembretes no dia a dia das medidas terapêuticas acordadas e motivação para a adoção de compor-tamentos saudáveis | o | o | o | o | o | o |
| Maior acesso aos cuidados de saúde, p. ex., colmatando os tempos de espera para tratamento, opções de cuidados mais completos ou de níveis mínimos | o | o | o | o | o | o |
| Maior competência e literacia em saúde, p. ex., através de uma melhor educação dos pacientes | o | o | o | o | o | o |

Outros benefícios das aplicações digitais de saúde para os pacientes (opcional). Texto livre

Na sua opinião, quais as principais vantagens das aplicações digitais de saúde (“aplicações com receita médica”) para os profissionais de saúde?

| Discordo totalmente | | Discordo | Não consigo decidir | Concordo | Concordo totalmente | Não sei |
| --- | --- | --- | --- | --- | --- | --- |
| Aquisição de novos pacientes | o | o | o | o | o | o |
| Maior satisfação dos pacientes | o | o | o | o | o | o |
| Economia de tempo a longo prazo por paciente através de ganhos de eficiência | o | o | o | o | o | o |
| Melhor assistência ao paciente | o | o | o | o | o | o |
| Maior sucesso do tratamento | o | o | o | o | o | o |
| Receita adicional | o | o | o | o | o | o |

Outros benefícios das aplicações digitais de saúde para os profissionais de saúde (opcional). Texto livre

Quais considera serem as maiores barreiras à prescrição de aplicações digitais de saúde?

| Discordo totalmente | | Discordo | Não consigo decidir | Concordo | Concordo totalmente | Não sei |
| --- | --- | --- | --- | --- | --- | --- |
| Falta de informação, p. ex., sobre as aplicações disponíveis, a adequação de determinada aplicação DiGA e/ou o processo de prescrição e comparticipação | o | o | o | o | o | o |
| Falta de evidências ou evidências insuficientes dos benefícios para os pacientes | o | o | o | o | o | o |
| Alto esforço inicial de formação e/ou familiarização para o pessoal médico | o | o | o | o | o | o |
| Necessidade de ajustes e adaptações dos processos de tratamento ou prática clínica existentes | o | o | o | o | o | o |
| Fraca integração ou compatibilidade com software e ferramentas de prática clínica existentes | o | o | o | o | o | o |
| Falta de apoio do fabricante em problemas ou questões técnicas | o | o | o | o | o | o |
| Aumento permanente do volume de trabalho de médicos e terapeutas devido a tarefas adicionais na assistência aos pacientes | o | o | o | o | o | o |
| Comparticipação desadequada de serviços médicos e terapêuticos concomitantes, p. ex., monitorização dos dados dos pacientes e resposta a questões de acompanhamento | o | o | o | o | o | o |
| Incerteza quanto à privacidade e segurança dos dados pessoais (de saúde) | o | o | o | o | o | o |
| Outras ambiguidades legais, p. ex., em relação ao risco de responsabilidade em caso de diagnóstico ou tratamento errado com base em dados da aplicação DiGA | o | o | o | o | o | o |

Outras barreiras à prescrição de aplicações digitais de saúde (opcional). Texto livre

O que é que poderá incentivá-lo a prescrever (mais) aplicações digitais de saúde?

| Discordo totalmente | | Discordo | Não consigo decidir | Concordo | Concordo totalmente | Não sei |
| --- | --- | --- | --- | --- | --- | --- |
| Mais informação sobre as aplicações disponíveis, a adequação de determinada aplicação DiGA e/ou o processo de prescrição e comparticipação | o | o | o | o | o | o |
| Maior comparticipação por serviços concomitantes, p. ex., através do código EBM, pagamentos de montante fixo ou extra-orçamentários | o | o | o | o | o | o |
| Oportunidade de experimentar aplicações digitais de saúde no âmbito de uma versão de teste | o | o | o | o | o | o |
| Recomendação de aplicações DiGA específicas por serviços de saúde | o | o | o | o | o | o |
| Integração de aplicações DiGA em contratos de cuidados de saúde integrados/contratos seletivos | o | o | o | o | o | o |
| Questões ou pedidos dos próprios pacientes | o | o | o | o | o | o |
| Troca direta com o fabricante de uma aplicação digital de saúde | o | o | o | o | o | o |
| Relatórios de experiências positivas de colegas, p. ex., em congressos médicos ou em revistas especializadas | o | o | o | o | o | o |
| Recomendação de uma ou mais aplicações DiGA por sociedades médicas/orientações científicas | o | o | o | o | o | o |

Outros fatores que possam incentivá-lo a prescrever aplicações digitais de saúde (opcional). Texto livre

Em que informações sobre as aplicações digitais de saúde é que está interessado? (possibilidade de múltipla seleção)

- - Quais as aplicações existentes?
  - Para que pacientes e indicações é que uma aplicação DiGA é adequada?
  - Quais os benefícios e riscos comprovados de uma aplicação DiGA?
  - Quanto custa uma aplicação DiGA e quais as companhias de seguros de saúde que comparticipam a despesa e como?
  - Como é que prescrevo uma aplicação DiGA?
  - Como é que os meus pacientes obtêm uma aplicação DiGA depois de eu a prescrever, e como é que a utilizam?
  - Como é que monitorizo a utilização de uma aplicação DiGA?
  - Outra(s) (por favor, especifique)
  - Não desejo receber qualquer informação adicional sobre as aplicações DiGA

Por favor, explique mais aprofundadamente a sua opinião sobre as aplicações digitais de saúde (opcional).

Texto livre

# Questões demográficas

Qual a sua especialização médica? Selecionar

Onde é que trabalha como médico ou terapeuta? Numa cidade/vila com...

- - mais de 500.000 habitantes
  - 100.001 – 500.000 habitantes
  - 20.001 – 100.000 habitantes
  - 5.001 – 20.000 habitantes
  - menos de 5.000 habitantes

Que tipo de local de trabalho melhor descreve a sua situação profissional?

- - Clínica
  - Consultório individual (sem outros colegas)
  - Consultório com outros colegas (p. ex., consultório conjunto ou de grupo)
  - Outro

É médico licenciado do serviço de saúde público ou profissional do setor privado?

- - Apenas médico licenciado do serviço de saúde público
  - Apenas profissional do setor privado
  - Ambos

Quantos médicos ou terapeutas trabalham no seu local de trabalho? Texto livre

Qual o seu nível de interação digital em contexto profissional? De 1 (sem qualquer interação digital) a 10 (com muita interação digital)

Qual a sua idade?

- menos de 26 anos
- 26 – 35 anos
- 36 – 45 anos
- 46 – 55 anos
- 56 – 65 anos
- mais de 65 anos

Qual o seu género?

- Masculino
- Feminino
- Outro
