# Supplementary material for: Mental Health Professionals’ Attitudes Toward Digital Mental Health Apps and Implications for Adoption in Portugal: Mixed Methods Study
Source: JMIR Hum Factors. 2023 Jun 2;10:e45949. doi: 10.2196/45949 (PMC10276319; doi:10.2196/45949)
Supplement: Multimedia Appendix 3 [file humanfactors_v10i1e45949_app3.docx]

| **Summary of modifications between survey questionnaires** | | |
| --- | --- | --- |
| **Dahlhausen et al. (2021)** | **Nogueira-Leite et al. (2023)** | **Observations** |
| Q1 | Q1 |  |
| Q2 | Q2 |  |
| Q3 | Q3 |  |
| Q4 | Q4 |  |
| Q5 | Q5 |  |
| Q6 | Q6 |  |
| Q7 | Q7 |  |
| Q8 | Q8 |  |
| Non-existent | Q9 | Question added by suggestion during survey adaptation |
| Q9 | Q10 |  |
| Q10 | Q11 | 'Additional income' and 'Acquisition of new patients' were removed as answer options due to non-applicability in the context. |
| Q11 | Q12 |  |
| Q12 | Q13 | Suppression of 'Higher reimbursement (…)', 'Opportunity to try (…)', and 'Recommendation of individual DiGA (…)' due to adapters considering them out of scope for the Portuguese context. Answer options #2, #3, and #5 were added due to relevance in the Portuguese context. |
| Q13 | Q14 |  |
| Q14 | Q15 |  |
| Q15 (Medical specialization) | Q01 | Q15 was substituted by Q01, where the choice was narrowed down to the relevant professions in the Portuguese setting |
| Q16 | Q02 |  |
| Q17 | Q03 | Added answer option 'Primary care services' due to relevance in the Portuguese setting |
| Q18 | Q04 | Answer option change due to a different organization of the Portuguese health system. |
| Q19 | Q05 |  |
| Q20 | Q06 |  |
| Q21 | Q07 |  |
| Q22 | Q08 |  |

| **Legend:** |  |  |  |  |  |  |  |  |  |
| --- | --- | --- | --- | --- | --- | --- | --- | --- | --- |
|  |  |  |  |  |  |  |  |  |  |
|  | Wording adapted to the Portuguese context. | | | |  |  |  |  |  |
|  | Question and answers' wording adapted to the Portuguese context. | | | | | |  |  |  |
|  | Answer option wording adapted to the Portuguese context. | | | | | |  |  |  |
|  | Other changes - please see the 'Observations' column. | | | | |  |  |  |  |
|  |  |  |  |  |  |  |  |  |  |
| **Remarks:** | |  |  |  |  |  |  |  |  |
|  |  |  |  |  |  |  |  |  |  |
| Question count was started at the first item of the questionnaire that posed a question (e.g., in Dahlhausen's questionnaire, the first question of point 4 corresponds to the below reported Q1).  All optional questions were replicated in Nogueira-Leite et al.'s questionnaire and, despite receiving numeration in the said questionnaire, are not considered in this matching table. | | | | | | | | | |
